# Arthroscopic partial meniscectomy in lateral meniscus tears: A surgical evaluation

**DOI:** 10.6026/973206300221865

**Published:** 2026-03-31

**Authors:** Chowdhury Iqbal Mahmud, Abu Zar M.D. Selimullah, Alinoor M.D, Yousuf Ali M.D, Ashraful Islam M.D

**Affiliations:** 1 Department of Orthopedic Surgery, Bangladesh Medical University, Dhaka, Bangladesh

**Keywords:** Arthroscopic partial meniscectomy, lateral meniscus tear, knee function, visual analog scale (VAS)

## Abstract

Traumatic lateral meniscal tears are common knee injuries, but outcome data on arthroscopic partial meniscectomy (APM) from low- and middle-income countries remain limited. This prospective interventional study was conducted at the Department of Orthopedic Surgery, Bangladesh Medical University, Dhaka, between March 2017 and September 2019 among 30 patients aged 18-50 years with arthroscopically confirmed lateral meniscal tears treated with APM and standardized rehabilitation. Pain, knee function, and disease-specific quality of life were evaluated using the Visual Analog Scale (VAS), Lysholm score, and WOMET score, while overall outcomes were graded using the Tapper-Hoover system. Postoperative outcomes improved significantly, with mean VAS decreasing from 8.63 ± 1.26 to 0.53 ± 0.71, WOMET increasing from 10.50 ± 3.57 to 83.70 ± 4.30, and Lysholm score improving from 7.40 ± 10.13 to 84.70 ± 12.73 (p < 0.001). Thus, arthroscopic partial meniscectomy resulted in substantial short-term improvements in pain, knee function, and quality of life, supporting its role for symptomatic traumatic lateral meniscal tears when repair is not feasible.

## Background:

Meniscal injuries are among the most frequent intra-articular knee lesions encountered in orthopedic practice. The estimated incidence is about 60-70 cases per 100,000 persons annually [1]. The medial and lateral menisci are crescent- shaped fibro-cartilaginous structures of the knee. They play key roles in load transmission, shock absorption, joint lubrication, proprioception and knee stability [2, 3]. Together, the menisci transmit approximately 50-75% of the tibiofemoral load in full extension. This proportion may increase to about 85% at 90°C of knee flexion [4]. Loss or resection of meniscal tissue reduces the tibiofemoral contact area. It increases contact stress within the knee joint. This process accelerates cartilage wear and degeneration. Consequently, the risk of early osteoarthritis increases [5]. Therefore, meniscal preservation has become a key principle of modern knee surgery. Meniscal tears commonly occur after twisting or pivoting trauma. These injuries are frequent in young and physically active individuals involved in sports or manual labor [6, 7]. Meniscal tears may also develop in middle-aged adults. In these patients, tears may result from degenerative changes or repetitive micro- injury. Historically, total meniscectomy was widely practiced. However, long-term follow-up studies showed unfavorable outcomes after complete meniscal removal. These outcomes include joint space narrowing, instability and premature osteoarthritis [8, 11]. These findings shifted the surgical approach toward meniscal preservation techniques. Arthroscopic partial meniscectomy removes unstable or irreparable meniscal fragments. A stable peripheral rim of the meniscus is preserved. This helps maintain the biomechanical function of the knee joint [9, 10].

The arthroscopic technique is minimally invasive and allows faster recovery. It also reduces postoperative morbidity and facilitates earlier return to function. Although arthroscopy is widely practiced, controversy remains regarding its effectiveness in degenerative meniscal tears. Several randomized trials in older adults with osteoarthritis reported no significant long-term benefit of APM. These studies compared APM with conservative management or sham surgery [12, 13 and 17]. However, these trials mainly evaluated degenerative tears rather than traumatic injuries. Traumatic meniscal injuries in younger patients represent a distinct clinical entity. They differ in pathophysiology, symptoms and prognosis [14, 15]. In such cases, APM remains an accepted treatment option when meniscal repair is not feasible. It often leads to relief of pain and improvement of knee function [16]. Recent observational studies and registry data from high-income countries report favorable short-term outcomes after APM for traumatic tears. These studies demonstrate significant improvements in pain and validated knee function scores [14, 15-16]. However, evidence from low- and middle-income countries remains limited. Delayed diagnosis, restricted arthroscopy access and variability in rehabilitation may influence outcomes [18]. In Bangladesh, arthroscopic surgery has expanded rapidly in tertiary centers during the past decade. This increase parallels the rise in sports- and trauma-related knee injuries among young adults [19, 20]. Therefore, it is important to evaluate the surgical outcomes of arthroscopic partial meniscectomy for traumatic lateral meniscus tears of the knee.

## Methods:

## Study setting and design:

This single-center prospective interventional study was conducted in the Department of Orthopedic Surgery at Bangladesh Medical University, Dhaka. The study period was March 2017 to September 2019.

## Study population:

The source population comprised all patients aged 18-50 years admitted during the study period with a clinical diagnosis of meniscal tear. Thirty consecutive patients had arthroscopically confirmed lateral meniscal injury. They were enrolled and followed prospectively through surgery and postoperative assessment.

## Inclusion criteria:

Eligible participants were 18-50 years old and had clinical features of meniscal pathology. A lateral meniscal tear was confirmed intraoperatively. All provided written informed consent for surgery and study participation.

## Exclusion criteria:

Patients were excluded if they had active knee infection or advanced degenerative osteoarthritis. Prior ipsilateral knee surgery was also exclusion. Multi-ligament injuries requiring alternative procedures were excluded. Systemic conditions interfering with rehabilitation or outcomes led to exclusion. Patients unable to comply with follow-up or scoring were excluded.

## Sample size and sampling strategy:

A consecutive sampling technique was used to minimize selection bias. All eligible patients meeting the inclusion criteria were enrolled during the study period. Enrollment continued until the target sample size of 30 was reached.

## Study procedure:

After consent, baseline demographic data and injury laterality were recorded. The mechanism of injury and symptoms were also documented. Symptoms included pain, difficulty squatting or walking, swelling, giving way and locking. Clinical evaluation included the McMurray test, Thessaly test, joint line tenderness and Apley's grinding test, each documented as positive or negative. All patients then underwent arthroscopic evaluation via standard anterolateral and anteromedial portals. After arthroscopic evaluation through standard anterolateral and anteromedial portals, the tear site (anterior horn, body, or posterior horn) and tear morphology (longitudinal/bucket-handle, transverse, or oblique) were documented. Arthroscopic partial meniscectomy was performed by selective resection of the unstable meniscal fragment while preserving a stable peripheral rim, as determined intraoperatively (Figure 1 - see PDF). Postoperative management followed institutional protocol. Early range-of-motion exercises were emphasized. Quadriceps activation and progressive weight-bearing were allowed as tolerated. Follow-up evaluations were performed at scheduled intervals and the final assessment was recorded at the last follow-up visit.

## Data collection:

Data were collected prospectively using standardized forms by trained study personnel and verified prior to analysis. Variables included demographics and injury characteristics such as side, mechanism and time to surgery. Clinical findings, arthroscopic details and radiologic healing categories were recorded. Pre- and postoperative VAS, WOMET and Lysholm scores were also collected.

## Laboratory and diagnostic evaluation:

Preoperative imaging followed institutional practice. Plain radiographs were obtained to exclude fractures and advanced degeneration. Magnetic resonance imaging was performed when clinically indicated to characterize meniscal pathology. Arthroscopy served as the diagnostic reference standard. Postoperative clinical assessments were performed in all patients. Radiological assessments were used when applicable. Meniscal healing was classified as 8-10, 10-12, or 13-16 weeks, based on departmental protocol.

## Outcome measures:

Primary outcomes were changes between preoperative and final postoperative assessments. Pain was measured using the Visual Analog Scale (0-10). Quality of life was measured using WOMET. Knee function was measured using the Lysholm Knee Scoring Scale. Global surgical outcome was graded using Tapper-Hoover criteria. Excellent and Good were classified as satisfactory. Fair and Poor were classified as unsatisfactory. Secondary variables were analyzed descriptively. These included operative timing (≤6 vs >6 months), tear morphology and associated injuries. Associated injuries included isolated lateral meniscus tears, lateral plus medial meniscus tears and lateral meniscus with other injuries.

## Statistical analysis:

Data were analyzed using SPSS version 25 (IBM Corp., Armonk, NY, USA). Categorical variables were summarized as frequency and percentage and continuous variables were expressed as mean ± standard deviation. Pre- and postoperative VAS, WOMET and Lysholm scores were compared using paired Student's t-tests. Effect sizes were reported as mean differences with 95% confidence intervals. A two-sided p-value < 0.05 was considered statistically significant. Missing data were minimal and were handled using complete-case analysis.

## Ethical considerations:

The study protocol was approved by the Institutional Review Board/Ethics Committee of Bangladesh medical university. Written informed consent was obtained from all participants prior to inclusion. Data was anonymized, stored securely and accessed only by authorized study personnel in accordance with institutional and national ethical standards.

## Operational definitions:

[1] Lateral Meniscus (LM) tear: Arthroscopically confirmed disruption of the LM substance.

[2] Arthroscopic Partial Meniscectomy (APM): Selective excision of the unstable or irreparable meniscal fragment, preserving a stable peripheral rim.

[3] Satisfactory outcome: Combination of Excellent and Good results per Tapper-Hoover grading.

[4] Unsatisfactory outcome: Combination of Fair and Poor results per Tapper-Hoover grading.

[5] Time to Union: Period (weeks) from surgery to clinical and radiologic confirmation of meniscal healing, categorized as 8-10, 10-12, or 13-16 weeks.

## Results:

Among the 30 patients included, the majority were between 26-35 years of age (50.0%), followed by 33.3% in the 18-25-year group and 16.7% aged 36-50 years (Table 1 - see PDF). The mean age of the patients was within the third decade of life and the youngest and oldest participants were 18 and 50 years, respectively. Most of the study subjects were male (90.0%), with only 10.0% being female. The right knee was affected more frequently (70.0%) than the left (30.0%). Regarding the mechanism of injury, road traffic accidents (33.3%) were the leading cause, followed by falls from height (26.7%), sports injuries (23.3%) and domestic injuries (16.7%). More than half of the procedures (53.3%) were performed after six months of injury, while 46.7% underwent surgery within six months of injury onset. Table 2 (see PDF) demonstrates the arthroscopic patterns of meniscal injury observed in the study. Isolated lateral meniscus (LM) injury was the most frequent finding, present in 86.6% of patients. Combined injuries were less common-6.7% had LM injury with other associated lesions and another 6.7% had concurrent medial meniscus (MM) injury. The body of the lateral meniscus was most commonly involved (46.7%), followed by the posterior horn (40.0%) and the anterior horn (13.3%). In terms of tear morphology, longitudinal (bucket-handle) tears were predominant (53.3%), while transverse and oblique tears accounted for 20.0% and 16.7%, respectively. Radiological evaluation showed that most tears healed within 10-12 weeks (56.7%). Healing occurred within 8-10 weeks in 16.7% of cases. Another 26.6% required 13-16 weeks. Table 3 (see PDF) presents the comparative analysis of preoperative and postoperative functional scores. The mean preoperative Visual Analog Scale (VAS) score was 8.63 ± 1.26, which significantly improved to 0.53 ± 0.71 postoperatively (p < 0.001). WOMET increased from 10.50 ± 3.57 to 83.70 ± 4.30. The Lysholm knee score improved from 7.40 ± 10.13 to 84.70 ± 12.73. Both changes were statistically significant (p < 0.001).

Figure 2 (see PDF) illustrates the distribution of preoperative clinical features among patients with lateral meniscal injury. Pain was a universal symptom, reported by 100% of participants. The next most frequent complaints were difficulty in squatting (83.3%), muscle wasting (73.3%) and difficulty in walking (70.0%). Intermittent swelling and a sense of giving way were also common, occurring in 66.7% and 60.0% of cases, respectively. Locking was present in 26.7% of patients, representing the least frequent symptom. Overall, pain and squatting difficulty were the predominant clinical features, highlighting the functional impairment associated with lateral meniscal tears. Figure 3 (see PDF) presents the distribution of positive clinical examination tests among patients with lateral meniscal injury. The McMurray test demonstrated the highest positivity rate, accounting for 33.3% of all positive findings. The Thessaly test followed with 31.0%. Joint line tenderness and Apley's grinding test each contributed 17.9%. Overall, dynamic clinical examination tests showed greater diagnostic positivity than static palpation-based tests in detecting lateral meniscal pathology in this study population during routine orthopedic clinical assessment settings.

## Discussion:

The present study demonstrated substantial improvements in pain, knee function and quality of life following arthroscopic partial meniscectomy. The mean VAS score decreased from 8.63 ± 1.26 to 0.53 ± 0.71 after surgery. WOMET improved from 10.50 ± 3.57 to 83.70 ± 4.30. The Lysholm score increased from 7.40 ± 10.13 to 84.70 ± 12.73. All improvements were statistically significant (p < 0.001). Based on Tapper-Hoover grading, 83.3% of patients achieved satisfactory outcomes. These findings support APM as an effective treatment option for symptomatic traumatic lateral meniscal tears. Similar improvements in early functional recovery after APM have been reported in previous studies [21]. The injury pattern in this cohort showed a predominance of isolated lateral meniscus tears (86.6%). Longitudinal or bucket-handle tears were the most common morphology (53.3%). The body of the meniscus was the most frequently involved site (46.7%). These patterns are consistent with twisting trauma in young and active individuals [2, 15]. Road traffic accidents were the leading mechanism of injury in this study (33.3%). This pattern reflects the regional trauma epidemiology in Bangladesh. Similar observations have been reported from tertiary care centers in the region [18, 19]. The results of this study are comparable with international reports. These studies have demonstrated rapid symptom relief and functional improvement after APM in traumatic meniscal tears. Structured rehabilitation also facilitates early return to activity [21]. In contrast, randomized trials in degenerative meniscal tears have shown different outcomes. These studies mainly involved older patients with osteoarthritis. They reported no significant long-term advantage of APM compared with non-operative or sham procedures [12, 13 and 17]. This difference highlights the important distinction between traumatic and degenerative meniscal injuries. Clinical examination tests also showed useful diagnostic value in this study. The McMurray test (93.3%) and Thessaly test (86.7%) showed high positivity rates. These findings support the role of dynamic clinical tests in detecting meniscal pathology [23, 24]. In many resource-limited settings, access to MRI may be restricted. Therefore, careful clinical examination remains important in the diagnostic evaluation. More than half of the patients underwent surgery more than six months after injury. Despite this delay, significant postoperative improvements were observed. These findings suggest that delayed APM can still provide favorable outcomes in selected symptomatic patients [7, 20]. Delayed presentation is common in low- and middle-income countries due to diagnostic and infrastructural barriers. The present findings therefore support the practical role of APM when meniscal repair is not feasible. However, the long-term consequences of meniscectomy remain an important concern. Experimental and clinical studies indicate that loss of meniscal tissue increases tibiofemoral contact pressure. This biomechanical change may accelerate cartilage degeneration and predispose to osteoarthritis [3, 11]. Therefore, meniscal preservation remains a fundamental surgical principle. Selective meniscectomy should only be performed for unstable or irreparable fragments. Preservation of a stable peripheral rim is essential. Early mobilization and quadriceps strengthening are also important components of postoperative rehabilitation [17, 18, 19, 20, 21- 22].

## Strengths and limitations:

Strengths include a prospective design and standardized data collection. Validated outcome measures (VAS, WOMET, Lysholm) were used. Tapper-Hoover grading provided global assessment. The cohort included only arthroscopically confirmed traumatic lateral meniscal tears. The study also contributes context-specific evidence from an LMIC tertiary center. Limitations include a small sample size (n = 30) and single-center design. There was no comparison group. Follow-up was short, limiting assessment of joint preservation. Radiologic categories were institutional rather than MRI-standardized. Selection factors (e.g., surgeon judgment, occupational demand) and rehabilitation adherence could also influence outcomes.

## Conclusion:

This prospective study included 30 adults with traumatic lateral meniscal tears treated with APM at BMU. Significant improvements in pain, function and quality of life were observed. Satisfactory outcomes were achieved in 83.3% of patients. These observations are consistent with reports supporting APM for traumatic, non-degenerative lesions when repair is not feasible and emphasize adjunctive, structured rehabilitation. Future studies should include larger, multicenter cohorts. Comparative designs are needed. Longer follow-up and standardized imaging are required to assess joint preservation and osteoarthritis progression.
